# Oromucosal Administration of Oxytocin: The Development of ‘Oxipops’

**DOI:** 10.3390/pharmaceutics16030333

**Published:** 2024-02-27

**Authors:** Dan Xu, Chunmei Lan, Juan Kou, Shuxia Yao, Weihua Zhao, Keith M. Kendrick

**Affiliations:** 1 The Clinical Hospital of Chengdu Brain Science Institute, MOE Key Laboratory for Neuroinformation, Center for Information in Medicine, University of Electronic Science and Technology of China, Chengdu 611731, China; xudan040901@gmail.com (D.X.); chunmeilan.uestc@outlook.com (C.L.); yaoshuxia@uestc.edu.cn (S.Y.); 2 Institute of Brain and Psychological Sciences, Sichuan Normal University, Chengdu 610066, China; koujuan@sicnu.edu.cn

**Keywords:** oromucosal administration, oxytocin, medicated lollipops, lingual spray, neuropeptides

## Abstract

The role of the hypothalamic neuropeptide oxytocin in influencing the brain and behavior has been the subject of widespread research over the last few decades due, most notably, to its reported involvement in promoting social cognition and motivation, reducing anxiety, and relieving pain. It is also increasingly being considered as an important therapeutic intervention in a variety of disorders with social dysfunction as a symptom. While, in recent years, studies in humans have administered oxytocin primarily via an intranasal route, since it may partly enter the brain directly this way via the olfactory and trigeminal nerves, there is increasing evidence that many of its functional effects can be peripherally mediated via increasing its concentration in the blood. This has opened up an oromucosal administration route as an alternative, which is beneficial since the oral consumption of peptides is problematic due to their rapid breakdown in the acidic environment of the gastrointestinal system. In this review we will discuss both the methodologies we have developed for administering oxytocin via lingual application and medicated lollipops, ‘oxipops’, in terms of increasing blood concentrations and the bioavailability of the peptide, and also their validation in terms of functional effects on the brain and behavior. While areas under the curve are significantly greater in terms of plasma oxytocin concentrations following intranasally relative to oromucosally administered oxytocin, with the estimated absolute bioavailability of the latter being around 4.4% compared with 11.1% for intranasal administration, the time to peak concentrations (around 30 min) and functional effects on the brain and behavior are broadly similar. We will also discuss potential therapeutic advantages of the oromucosal administration of oxytocin in different clinical contexts and its wider application for other peptides which are increasingly being developed for therapeutic use.

## 1. Introduction

The neuropeptide oxytocin is synthesized by cells in the hypothalamic paraventricular and supraoptic nuclei and transported to the posterior pituitary for release into the blood, where it is most well-known for acting on smooth muscle to stimulate uterine contractions during labor and milk-ejection from the breast [1]. However, there are also extensive projections from hypothalamic oxytocin neurons to many parts of the brain which are important for the control of social cognition, motivation, and a number of other behavioral and physiological functions [2,3,4]. In recent years, there has therefore been considerable interest in the therapeutic use of oxytocin administration, particularly for social dysfunction in disorders such as autism [5,6,7,8]. There is currently no approved medication-based treatment for autism and, while clinical trials have reported some positive effects for improving social symptoms, factors such as the dose magnitude and frequency as well as the route of administration may all be important [5,6,7,8]. Furthermore, oxytocin-based treatments may also have beneficial effects for reducing anxiety and depression [9] and for relieving pain [10]. To date, all randomized clinical trials have chosen to use an intranasal route for the chronic administration of oxytocin and have not considered the possibility of using some form of oral administration as an alternative. For pediatric and geriatric patient populations, oral administration would represent a considerable advantage in terms of tolerability and compliance for long periods of treatment. This review firstly details the likely mechanisms whereby oxytocin administration can produce functional effects on the brain and behavior, which demonstrate the potential to use an oral instead of an intranasal route, and, secondly, this review details how an effective oromucosal administration strategy has been developed, which opens up the possibility for orally administered oxytocin to be used in future clinical trials.

## 2. Mechanisms Whereby Oxytocin Administration May Produce Functional Effects

Oxytocin is a small nine-amino acid peptide with a molecular weight of 1007.2 Daltons and which binds to a single seven-transmembrane domain G protein-coupled receptor. Oxytocin is hydrophilic (logP < 1 for lipophilicity), non-polar, non-charged, and both heat- and UV light-sensitive [2,11]. Its primary clinical use to date has been in the induction of labor and control of post-partum hemorrhage by its intravenous infusion or intramuscular injection through its actions on receptors in uterine smooth muscle [1]. However, there has been increasing interest in administering oxytocin to influence brain and behavioral functions. Small hydrophilic peptides, such as oxytocin, have difficulty crossing the blood–brain barrier, and this has led to its administration by intranasal spray in humans as a method for allowing it to penetrate into the brain to produce its functional effects [3,4,5,6,7,8,9,10]. Using animal models, studies have primarily identified the olfactory and trigeminal nerves as the routes whereby intranasally administered oxytocin can enter the brain, although, additionally, a large amount of the peptide is absorbed by the nasal vasculature and enters the peripheral circulation (see [4]). Oxytocin has receptors in numerous peripheral organs including the cardiovascular and gastrointestinal systems as well as the dorsal root [12] and trigeminal ganglia [13]. Thus, it is possible that peripheral increases in oxytocin may have profound effects on vagal projections to the brain to influence behavior directly without entering the brain, but it could also potentially act via this route to promote endogenous oxytocin release within the brain. However, the potential for oxytocin to produce functional effects on the brain and behavior via these peripheral receptors has been largely overlooked. Additionally, it is possible that increased concentrations of oxytocin in the peripheral circulation may cross the blood–brain barrier to enter the brain after binding to receptors for advanced glycation end-products (RAGE) [14]. This recent discovery has added a further level of complexity to the field, and although experiments using genetically modified animals lacking an expression of RAGE have suggested that this can influence the ability of peripherally administered oxytocin to produce effects on the brain and behavior, it is still unclear whether RAGE might play a similar role in humans.

While the question of the precise mechanism whereby intranasally administered oxytocin might enter the brain, or otherwise influence its function, is still not completely established, what has become increasingly clear is that many of its observed functional effects do indeed appear to depend on increased peripheral concentrations rather than following direct entry into the brain. Three lines of evidence support this conclusion. The first is from a number of intranasal administration studies reporting that functional changes in the brain are associated with peripheral concentration changes [15], the second is from animal model and some human studies demonstrating that behavioral and brain effects can occur via routes of administration where direct entry of the peptide into the brain cannot occur (i.e., intravenous, subcutaneous, and intraperitoneal) (see [4]), and the third, and most recent, line of evidence, is from a study showing that neural effects following intranasal administration can be prevented using pre-treatment with a vasoconstrictor (otrivine), which considerably reduces increased peripheral blood concentrations but should not have any effect on preventing direct entry into the brain. This electroencephalography study found that the effects of a 24 IU intranasal oxytocin dose on increasing cross-frequency coupling in the brain between beta and delta frequencies (which may reflect neural effects of oxytocin on attention) could be almost completely eliminated by pre-treatment with the vasoconstrictor. Additionally, the neural effects of intranasal oxytocin in this study, in the absence of vasoconstrictor treatment, were also associated with increased concentrations of the peptide in the blood [16]. This latter finding effectively demonstrates that, while there may be some functional effects of intranasal oxytocin mediated via direct entry to the brain via the olfactory and trigeminal nerves, it is its increased concentrations in the peripheral circulation which may be of most importance [4].

Taken together, the above lines of evidence demonstrate that functional and potentially therapeutic effects of exogenously administered oxytocin can be achieved via routes which only increase peripheral vascular concentrations without directly entering the brain. For repeated dosing regimens in humans, particularly in pediatric or geriatric populations, intravenous, intramuscular, or subcutaneous injections may not be the best tolerated and this raises the question of whether an oral administration route of some kind might be optimal. While pills or capsules containing oxytocin may have some potential for allowing its absorption into the blood via the gastrointestinal tract, its acidic environment and the presence of peptidases render this method suboptimal [17]. Furthermore, swallowing pills can be problematic for both young children and geriatric patients. For example, for a related neuropeptide, vasopressin, its synthetic analogue desmopressin has been estimated to only achieve a bioavailability from 0.08 to 0.16% when given orally as a pill to be swallowed [18]. One animal study has reported that the absorption of oxytocin into the blood via the gastrointestinal tract can be enhanced following pre-treatment with proton pump inhibitors to reduce the acidic environment [19], but this is not a particularly viable option for long-term treatment in humans, especially in pediatric populations. On the other hand, oromucosal administration where oxytocin can be absorbed by blood vessels in the mouth is far more promising, provided that the method used can permit the peptide to remain in the mouth for a sufficient duration to allow maximum absorption into the blood and be well-tolerated by patients. The main challenge has, therefore, been to develop viable methods for the oromucosal administration of oxytocin which could overcome potential drawbacks of its physicochemical properties and be potentially used in clinical practice, particularly for pediatric and geriatric populations.

## 3. Oromucosal Administration of Peptides

**Figure 1:**
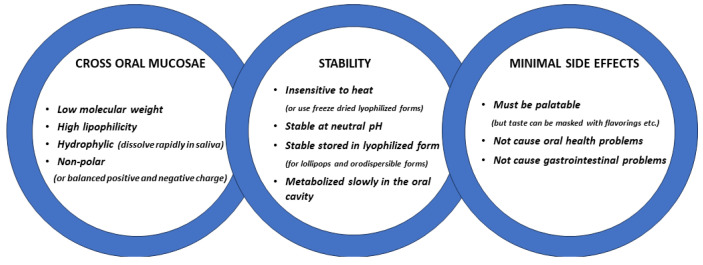
Ideal features for potential peptide-based drugs suitable for oromucosal administration.

The oral cavity is easily accessible for the administration of peptides and the main issue is to ensure that they remain in the mouth for a sufficient time to be absorbed into the blood via the mucosae. The two main pathways by which molecules diffuse passively across mucosal membranes are paracellular (passage between adjacent cells, i.e., intercellular) and transcellular (passage through cells) routes [11,20]. The paracellular diffusion route predominates for most lipophilic and hydrophilic molecules when they cross mucosal membranes with two types of pathways within the intercellular space; a hydrophobic pathway through the lipid bilayers and a parallel hydrophilic one along the narrow aqueous regions associated with lipid polar head groups. As the lipophilicity of a molecule increases, the propensity for its diffusion across the epithelial barrier also increases, although the main influence on diffusion is molecular weight, and other factors such as charge can also be important for non-polar molecules to cross the epithelial layer more effectively [11] (see Figure 1). A key advantage of the oromucosal administration of substances, as opposed to oral administation using pills, is that they can enter the blood directly and thereby reduce first-pass metabolism depletion by the liver and gut [20].

The complex structure of peptides represents a challenge for their delivery via a mucosal route, as they have poor physicochemical stability, high molecular weight, and, often, low lipophilicity resulting in problems permeating mucosae and entering the blood [21]. The buccal and sublingual regions of the oral cavity are the main targets for oromucosal administration since they are non-keratinized and highly vascularized, and sub-lingual tissue has a limited number of epithelial cell layers [22]. Currently, there are three main approaches to the oromucosal delivery of drugs: orodispersal using fast-dissolving tablets, films, or gels; intra-oral sprays; and drugs incorporated into lollipops, gum, or chocolate. Of these three approaches, the most often clinically used to date has been the orodispersal method using tablets, films, or gels which dissolve in saliva in the oral cavity and do not require water for swallowing (see [23] for review). Originally, for example, an oromocosal tablet containing oxytocin was used to help induce labor [24,25], although intravenous administration is more effective and now preferred. Currently, only a single therapeutic peptide, desmopressin, a synthetic analogue of arginine vasopressin, is approved and used for oromucosal administration in the treatment of enuresis nocturna [26]. Desmopressin is approximately 10 times more potent as an anti-diuretic agent than vasopressin itself [27], although it has a similar molecular weight (1069 vs. 1084 Daltons). When administered sublingually as a lyophilized tablet, its bioavailability is reportedly only 0.25% [28] in contrast to 5–10% when administered via an intranasal route [29]. Desmopressin’s effects on life-threatening hyponatremia are particularly associated with intranasal administration, which has led to the initial approval for its use intranasally being withdrawn by the FDA [30]. Therefore, desmopressin containing minitablets with a mucoadhesive film have been developed for buccal delivery which do not require swallowing, can improve compliance [31], and have a very low incidence of hyponatremia [28]. More recently, orodispersible films and gels have been developed which, when placed on the tongue or cheek, dissolve rapidly to release drugs into the oral cavity [23,32]. However, the substances incorporated into orodispersal mediums may be limited in terms of their concentration, released too fast, and/or swallowed before maximum absorption into the blood occurs, and compliance and tolerance in pediatric and geriatric populations in particular may be more problematic. Thus, the two other main methods for the oromucosal delivery of peptides and other substances, using intraoral sprays and medicated candies, may offer some advantages.

### Development of Oromucosal Oxytocin Administration Using Lingual Spray and Medicated Lollipop ‘Oxipop’ Methodologies

**Figure 2:**
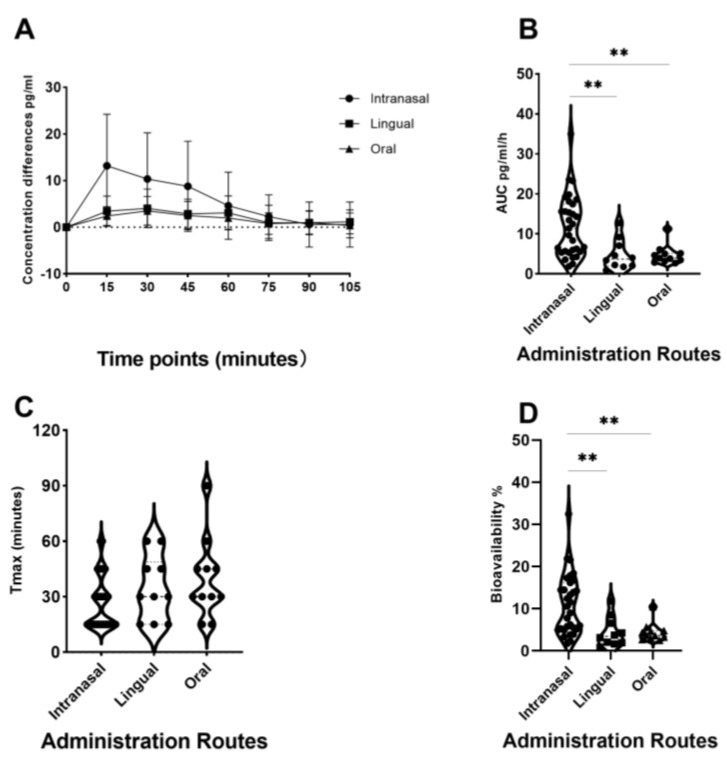
Comparison of pharmacodynamic profiles of oxytocin concentrations in blood following intranasal, lingual, and oral (medicated lollipops, ‘oxipops’) administration of 24 IU oxytocin. (**A**) Changes in plasma oxytocin concentrations following 24 IU dose administration, means ± SD. Violin plots and individual data points (black dots) showing (**B**) areas under the curve (AUC) (**C**), Tmax, and (**D**) absolute bioavailability. ** *p* < 0.01, for post-hoc tests following significant (*p* < 0.001 for both AUC and bioavailability) one-way ANOVAs (Kruskall–Wallis). Data are taken from [16,33,39]. For bioavailability calculations, plasma concentrations of oxytocin following intravenous infusions are taken from a publication by another group who have made their data publically available [15].

Given that oxytocin is commercially available as an intranasal spray and licensed for use as a lactation enhancer in post-partum women (e.g., Sichuan Defeng, Pharmaceutical Co., Ltd., Chengdu, China), a first step into assessing its efficacy when given orally was to utilize a spray for administration by lingual application. The spray contains oxytocin acetate at a concentration of 4 IUs per 0.1 mL puff and is dissolved in 0.9% saline and stabilized using glycerol. Placebo sprays are identical in composition with the exception that the peptide is excluded. The same protocol was therefore adopted as for intranasal administration with 6 × 4 IU doses being self-administered under and on top of the tongue and with each spray being given at 30 s intervals to match the intranasal protocol (i.e., a total administration duration of 3 min). To maximize absorption, subjects were required to keep the spray in their mouth without swallowing until they self-administered the next dose [33,34,35]. Initially, in these studies, a pharmacokinetic assessment was carried out in 10 male adult male subjects with blood samples being taken at 15 min intervals before and for 105 min after the lingual administration. The profile of the altered blood concentrations, area under the curve (AUC), Tmax, and bioavailability measures are shown in Figure 2A–D and contrasted with those for 24 IU intranasal oxytocin. The absolute bioavailability was calculated using data for measurements of oxytocin concentrations in the patients’ blood following a 10 min intravenous infusion of 10 IU taken from [15], with repeated samples being taken over a similar time course. For lingual administration following a 24 IU dose, the AUC for the altered plasma oxytocin concentrations was significantly less than that for the intranasal administration (mean ± SD 4.79 ± 3.38 (*n* = 10) vs. 11.93 ± 7.68 pg/mL/h (*n* = 27), *p* = 0.008), as was the absolute bioavailability (4.45 ± 3.51% vs. 11.07 ± 7.12%, *p* = 0.008). The calculated bioavailability for the intranasal administration was very similar to that using data from a previous published paper where a 40 IU dose of oxytocin was used (10.59 ± 7.95%—from [15]). For the time to reach maximum concentrations in blood (Tmax), the lingual administration was 34.5 ± 17.4 min compared with 26.61 ± 14.34 min for the intranasal administration and did not differ significantly (*p* = 0.159). This obviously contrasts with profiles following intravenous infusions, where blood concentrations rise immediately and sharply [15].

However, lingual sprays where subjects are required to deliberately retain the liquid in their mouth for a period of time to maximize absorption are also clearly not optimal in terms of patient compliance, particularly in pediatric subjects. In recent years, medicated chocolate and candy lollipops have also been increasingly used to administer a variety of substances ranging from vitamin and mineral supplements to antibiotics and pain killers (see [36,37,38]). Typical lollipop formulations tend to be either hard or soft forms of sugar-free candy, with the substance for medication being incorporated into the candy during the heating stage of its formation. The candy lollipops in particular have a number of advantages for drug administration via oromucosal absorption since, during sucking, they are in constant close contact with the roof of the mouth and tongue and are pleasant to eat, especially for children. The two main disadvantages of such medicated lollipops are that, firstly, since a heating process is used in their conventional manufacture, this precludes using them for heat-sensitive drugs and, secondly, if the drug used has a strong bitter or unpalatable taste, this can also be a problem. Thus, the conventional strategy of incorporating drugs into the whole of a lollipop using a heating process is not suitable for peptides, which are typically heat-sensitive and would therefore degrade using such a strategy. To overcome this problem, the novel step was taken of freeze-drying oxytocin onto the surface of the lollipop to create ‘oxipops’ rather than trying to incorporate it into the whole lollipop. This had the dual advantage that current commercial sugar-free lollipops could be utilized and that it maximized the peptide being dissolved by the saliva as the child or adult sucked the lollipop with the whole dose being released slowly without the requirement that all of the lollipop needed to be consumed, since the oxytocin dose was only on the surface. These commercial lollipops typically use xylitol as a sweetener, which is widely accepted to be a safe form of low-calorie sweetener that does not cause insulin spikes and can actually promote oral hygiene and even immunocompetence [39]. Clearly, both children and adults may exhibit distinct taste preferences which could potentially influence compliance, although this can be overcome through the use of different flavor alternatives (current options have included orange, grape, pineapple, and pear). Oxytocin does not have a distinctive taste per se, so there is no problem in this respect.

**Figure 3:**
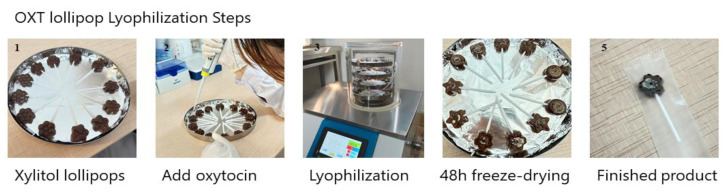
Method (left to right) for producing sugar-free (xylitol) lollipop candies with oxytocin acetate (OXT) freeze-dried on one surface of the lollipop (‘oxipops’).

The ‘oxipop’ protocol involves firstly dissolving different peptide concentrations in volumes from 0.1 to 0.2 mL of 0.9% saline (with glycerol added as a preservative) and then freeze-drying the lollipops using two standard cycles lasting around a total of 48 h in a commercial freeze-dryer [40]. The resultant ‘oxipops’ are then sealed and placed in a fridge (2–8 deg C) until required (see Figure 3). The ‘oxipops’ can be stored stably for 3–6 months although possibly for longer periods. The freeze-drying process causes the formation of a white residue on the surface of the lollipop and so it is important for the purposes of blinding in placebo-controlled experiments to also produce placebo lollipops using application of the saline and glycerol solution without the peptide and using the same freeze-drying process. Sucking the ‘oxipops’ for a total of 3 min can achieve similar increases in plasma concentrations of oxytocin as does direct administration of the same concentration over the same time period using lingual sprays (see Figure 1). The AUC is 4.74 ± 2.41 pg/mL/h (*n* = 11, *p* = 0.004 vs. intranasal and *p* = 0.97 vs. lingual), with an estimated bioavailability of 4.4 ± 2.23% (*p* = 0.008 vs. intranasal and *p* = 0.97 vs. lingual), thereby confirming in vivo that a similar concentration of oxytocin is being released into the oral cavity and absorbed into the blood as with a direct lingual spray using the same dose. The Tmax was 39.5 ± 21.5, which is not significantly different than that for lingual or intranasal administration (Kruskall–Wallis ANOVA, *p* = 0.095).

**Figure 4:**
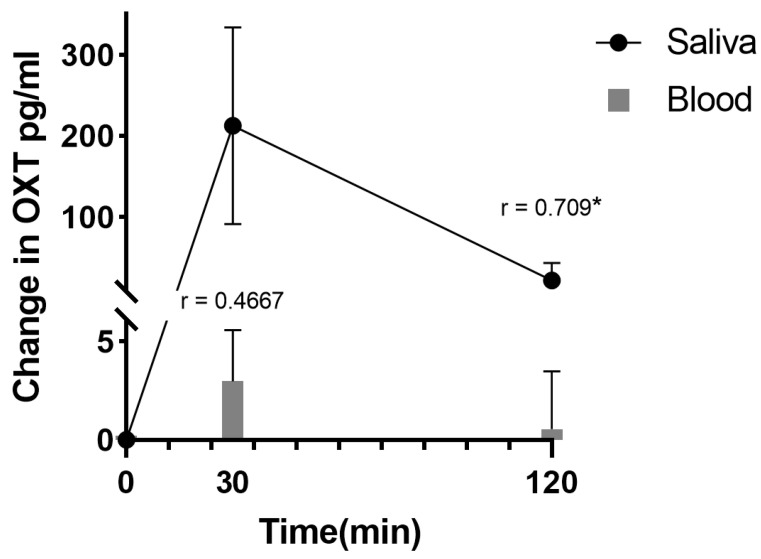
Changes in saliva (line) relative to plasma (histograms) concentrations of oxytocin (OXT) 30 min and 2 h following administration of 24 IU oral oxytocin by medicated lollipop, ‘oxipops’. Means ± SDs are plotted. Correlations (Spearman) between changes in saliva and plasma concentrations are also indicated. * *p* < 0.05.

As expected, the saliva concentrations of oxytocin increased considerably after sucking the ‘oxipop’ for 3 min (from a baseline mean ± SD of 8.70 ± 4.58 pg/mL to = 231.8 ± 116.9 pg/mL, *p* < 0.001 30 min after sucking the ‘oxipop’) and remained significantly elevated even 2 h afterwards (30.54 ± 19.12 pg/mL *p* < 0.001) (see Figure 4 and [39]). Interestingly, the pharmacodynamic profiles were similar to those reported for saliva oxytocin concentrations following intranasal administration, reinforcing suggestions that, with the use of nasal sprays, a large proportion of the peptide leaks down into the oral cavity, resulting in prolonged increases in saliva for 2–7 h [41,42,43,44]. While there was not a good relationship between the saliva and plasma concentrations of oxytocin at baseline (Spearman r = −0.212, *p* = 0.5563), in agreement with previous studies [5,45], following oral oxytocin administration using ‘oxipops’, this became positive by 30 min (r = 0.467, *p* = 0.174) and by 2 h was significant (r = 0.709, *p* = 0.0216 = see Figure 3), indicating that alterations in the saliva concentrations post administration were a reasonably accurate guide to likely increases in blood. At this stage it is not known if these peripheral changes following the oromucosal administration of oxytocin also result in increases in cerebrospinal fluid concentrations similar to those reported following intranasal oxytocin [46].

## 4. Neural and Behavioral Effects of Oromucosal Administration of Oxytocin

A number of proof-of-concept studies have been conducted in human participants where the efficacy of single doses of oxytocin given by intranasal, lingual, and oral ‘oxipop’ routes was compared using both brain and behavioral measures. The first of these studies used a neural biomarker for the effects of intranasal oxytocin in adult male subjects, where it has been shown to decrease the responses of the amygdala to emotional faces [47,48], although it has been shown to increase them in females [49]. Findings demonstrated that the lingual administration of oxytocin increased rather than decreased amygdala responses in males and also tended to increase them in females. Additionally, lingual oxytocin increased the responses of the putamen (a brain reward region) in both males [34] and females [50], whereas intranasal oxytocin only did so in females. However, overall, lingual and intranasal oxytocin both appeared to influence neural responses to emotional faces, although with some route-dependent differences which might possibly have been due to the fact that increased oxytocin concentrations were greater after intranasal, compared to lingual, administration. Additionally, 24 IU of both intranasal and lingual oxytocin produce a similar facilitation of the perceived pleasantness of soft, caress-like touch, but not of medium pressure touch, as well as increased responses in brain reward (orbitofrontal cortex) and social cognition (superior temporal gyrus) processing regions [33]. Finally, a series of studies investigating the effects of oxytocin on top-down compared with bottom-up attention control found equivalent effects of 24 IU doses administered intranasally, lingually, and via ‘oxipops’ on top-down attention control, particularly in relation to social stimuli but also to some extent for non-social ones, using an anti-saccade task [35,40]. In this task, subjects were instructed to direct their eye movement (saccade) from a central point either towards (prosaccade) or away (anti-saccade) from a social or non-social stimulus. In general, subjects found it hard to look away from the social stimulus (anti-saccade), made more errors, and were slower to respond relative to when asked to look towards stimuli (pro-saccade). All three routes of oxytocin administration equivalently increased the number of errors subjects made when instructed to look away from stimuli and increased the time taken to look away from social stimuli. As such, this is a very sensitive task for measuring the facilitatory effects of oxytocin on the top-down control of attention and particularly social attention, which has important therapeutic potential in disorders where individuals exhibit impaired attention to social cues.

## 5. Discussion and Future Potential Developments and Applications

The overall conclusion from the current review is that the oromucosal administration of oxytocin either using lingual spray or medicated lollipops (‘oxipops’) can produce reliably increased concentrations of the peptide in the peripheral vasculature, and similar functional effects were observed as for the same dose administered intranasally. The timing of peak concentrations of oxytocin were similar for intranasal, lingual spray, and oral ‘oxipop’ administration routes at around 30 min. On the other hand, the estimated bioavailability of the peptide delivered via the oromucosal routes was around 4.5% compared with around 11% after intranasal administration. The difference in bioavailability between the intranasal and oromucosal routes could reflect greater absorption of the peptide into blood across the nasal mucosa, but since concentrations were additionally greatly increased in saliva for a long duration after intranasal administration, it might also reflect combined absorption across both nasal and oral mucosae.

While there is still the possibility that some functional effects of the intranasal administration of oxytocin might be mediated via its direct entry into the brain via the olfactory and trigeminal nerves, which could not be achieved by an oromucosal route, evidence to date supports the conclusion that many functional effects are mediated by increased blood concentrations [4,16,33,34,35,40,50]. Most notably, in a recent study where the ability of intranasally administered oxytocin to enter the peripheral circulation was greatly reduced by the pre-administration of a vasoconstrictor (otrivine), the functional effects of a 24 IU intranasal oxytocin dose were almost completely eliminated. This latter finding effectively demonstrated that, while some functional effects of intranasal oxytocin might still be mediated following direct entry to the brain via the olfactory and trigeminal nerves, the increased concentrations in the peripheral circulation are likely to be of the most importance functionally. As such, this renders the potential therapeutic efficacy of the oromucosal administration of oxytocin to be at least equivalent to that of intranasal administration, although possibly actually improved since the oromucosal route offers considerable practical advantages (see [26]). The small size of nasal passages in young children and problems due to nasal blockages during colds or allergic conditions, coupled with the necessity for administration being given by caregivers or medical staff rather than by the individual themselves, makes the intranasal administration of oxytocin, or any other substance, potentially problematic, particularly in pediatric and geriatric populations. Using the novel freeze-drying protocol for oxytocin on the surface of sugar-free lollipops to create ‘oxipops’ also makes dissolving it into the mouth both rapid and prolonged (the ‘oxipop’ only needs to be sucked for around 3 min) and has the advantage of being perceived as extremely pleasant by the recipient. A potential drawback of the oral administration of oxytocin, similar to intranasal administration, is that a large amount of the peptide is swallowed and enters the gastrointestinal system. No studies to date using either acute or chronic doses of intranasal oxytocin have reported any significant gastrointestinal side effects and similarly acute doses of oromucosally administered peptide have not been reported to cause problems. Possibly, this reflects the rapid metabolism of oxytocin in the acidic conditions present in the gastrointestinal system.

To date, only the most soluble form of oxytocin, oxytocin acetate, which is soluble up to 5 mg/mL, has been used for oromucosal administration. Evidence suggests that, following administration via an ‘oxipop’, saliva concentrations remain elevated for at least 2 h and probably much longer given observations from intranasal administration studies. Thus, at this stage, using more stable forms of the peptide may not be that advantageous, although recent studies using the intranasal administration of a more stable analog suggest that the optimum dose required might be considerably reduced (TTA-121 [7]). Therefore, there may be some advantages to using formulations designed to enhance the stability of the peptide. Given that the mucosal barriers in the mouth do reduce the effective absorption of native oxytocin into the peripheral blood stream [11], there may be advantages in producing formulations with an improved ability to permeate the oral mucosae and thereby more efficiently increase blood concentrations. For example, penetrant enhancers such as fatty acids, surfactants, cholates, lauric acid, and alcohols or emulsions in the form of deformable liposomes and nanoparticles have been used as well as materials such as polyacrylic acid, chitosan, cellulose derivatives, alginate, and hyaluronic acid (see reviews [51,52,53]). However, the main issues limiting the passage of oxytocin across mucosal barriers would appear to be its high molecular weight and low lipophilicity [11], and so the development of peptidomimetic drugs which can circumvent these issues may be a more promising future approach.

Another possibility is that ‘oxipops’ could additionally contain beneficial concentrations of essential vitamins and minerals. Lollipops containing vitamin C or D and zinc are, for example, currently commercially available for the potential facilitation of immunocompetance. Zinc concentrations are often low in autistic children [53,54,55] and are associated with symptom severity [56]. There has also been research indicating that oxytocin may become more stable after binding to zinc and that low levels of zinc may actually impair the ability of oxytocin to bind to its receptor [57,58].

At this stage, only the effects of single doses of oromucosally administered oxytocin have been assessed on increased concentrations in blood and saliva and on neural and behavioral effects. Potential therapeutic effects of oxytocin in relation to autism or other disorders are likely to require chronic periods of administration over periods of weeks or months and, to date, this has only been investigated using intranasal administration [5,6,7,8,47]. Clearly, establishing potential therapeutic effects of chronic oxytocin using oromucosal administration is a priority for future research.

The precise mechanism whereby increased peripheral concentrations of oxytocin following oromucosal administration might influence the brain and behavior is also unclear, just as it is for intranasal administration. At this point, the most likely mechanism is via acting on receptors in the vagal system to both influence neural circuitry controlling behavioral functions and possibly additionally an increased release of endogenous peptide within the brain. However, there still remains the possibility that some oxytocin could be transported from the blood into the brain after binding to RAGE and crossing the blood–brain barrier [14]. It will also be important to establish whether there are similar inverted-U dose-dependency issues for oromucosal administration similar to intranasal oxytocin [7,48]. Additionally, for chronic dosing protocols, studies will need to determine whether an infrequent every-other-day dosing strategy produces improved functional effects compared to a more frequent one where doses are given once or twice a day in line with recent findings for repeated doses of intranasal oxytocin [5,47]. However, given increasing evidence for potential therapeutic effects of oxytocin in a range of disorders, including autism, anxiety, depression, schizophrenia, and chronic pain [5,6,7,8,9,10], the potential for using oromucosal administration as an intervention will be important to establish in future clinical trials.

While the current review has specifically focused on the development of the oromucosal administration of oxytocin to influence brain and behavioral functions, particularly through the use of a freeze-drying protocol where the peptide is simply present on the surface of a lollipop, this strategy has a wider potential application for the administration of oxytocin in other contexts. While intravenous oxytocin infusions and intramuscular injections are used routinely in developed countries for the management of labor and post-partum haemorrhage, in under-resourced, third-world countries, administration via these routes requires skilled medical staff and can be less available and affordable. Post-partum haemorrhage occurs after around 10% of births and is a major contributor to maternal mortality (35–50% of cases) [59,60]. Additionally, given that oxytocin is more stable in its dry form than in solution, there may be advantages for longer-term storage in hot climates with a limited availability of refrigeration. Indeed, one study has reported that lyophilized oxytocin is stable for 12 months when stored at 40 deg. C whereas, in solution, it degrades significantly after a month [60]. Freeze-dried oxytocin for oromucosal administration may therefore offer both practical and cost advantages.

While oromucosal administration using medicated candies is still a relatively novel strategy and has yet to be fully established for other suitable candidate peptide or non-peptide drugs, it may open up future possible avenues for administering either acute or long-term medication to individuals where other routes are less well-tolerated. In recent years for example, there has been a considerable growth in the number of peptide-based therapeutic drugs, with currently > 80 having been approved for use [61]. Peptide drugs offer several advantages over small molecules, including a heightened target specificity and potency and reduced side effects, although few have been developed for oromucosal administration. A full consideration of the current numerous peptide-based drugs, other than oxytocin, is beyond the scope of the current review and has been covered in another publication (see [61]). As outlined in Figure 1, the two obvious potential limitations for the oromucosal administration of peptide-based drugs as opposed to small molecules are that high molecular weights may result in poor penetration through the oral mucosae and that they are often metabolized too rapidly. It is therefore likely that, in a number of cases, more stable peptidomimetic drugs with a smaller molecular weight would have to be developed in order for them to be used with oromucosal administration. There is also the potential issue of peptide-based drugs being unpalatable or causing gastrointestinal problems following their ingestion. A further important issue will be to ensure the long-term stability of drugs for storage prior to use although refrigeration, storage in the dark, and vacuum-sealing should be effective in most cases. Where the refrigerated storage of heat-sensitive compounds is not readily available, they may still be more stable in a dry lyophilized form than in solution, although the addition of chemical stabilizers is another option.

Future developments in technology for the intraoral delivery of drugs may additionally provide alternatives to medicated lollipops. For example, advances in pharmaceutical technology and polymer science are increasingly allowing the manufacture of new types of intraoral administration using sprays, films, tablets, wafers, chewing gums, buccal patches, and bioadhesive tablets to deliver a wide variety of both non-peptide and peptide drugs (see [23,62]). For example, in terms of the oral delivery of peptides, an aerosol droplet application for an insulin delivery system based on a colloidal liquid formulation has recently been developed (Oralin^®^, Generex Biotechnology Corp., Toronto, ON, Canada) which utilizes a mixture of surfactants as stabilizers and permeation enhancers. This was found to be effective for the rapid oral absorption of insulin and control of glucose levels, and diabetic patients were more compliant than when using the traditional subcutaneous injection method [63]. It is therefore likely that the oromucosal administration of medications will become more widely established in future.

## 6. Conclusions

In conclusion, since many therapeutic effects of oxytocin appear to be due increasing its peripheral concentrations, oromucosal delivery, particularly using a novel medicated lollipop, ‘oxipop’, approach, may be an effective way of optimally increasing blood concentrations and offers considerable practical advantages over intravenous or intranasal routes, most notably for chronic use in pediatric and geriatric populations but possibly also for the management of labor and post-partum haemorrhage in some contexts. While the resultant absolute bioavailability using the native peptide is relatively low, concentrations remain significantly elevated for at least 2 h or more. Importantly, robust functional effects on the brain and behavior using acute doses have been observed using a number of different paradigms, although future research is needed to assess dose-dependency and dose-frequency effects. Additionally, using techniques to increase the stability of the peptide and penetrant enhancers to aid its absorption into circulation may be able to improve functional effects, particularly in the context of chronic administration regimes.

## Data Availability

No new data in this paper.

## References

[1] Hermesch A.C., Kernberg A.S., Layoun V.R., Caughey A.B. (2023). Oxytocin: Physiology, pharmacology, and clinical application for labor management. Am. J. Obstet. Gynecol..

[2] Gimpl G., Fahrenholz F. (2001). The oxytocin receptor system: Structure, function, and regulation. Physiol. Rev..

[3] Kendrick K.M., Guastella A.J., Becker B. (2017). Overview of human oxytocin research. Curr. Top. Behav. Neurosci..

[4] Yao S., Kendrick K.M. (2022). Effects of Intranasal Administration of Oxytocin and Vasopressin on Social Cognition and Potential Routes and Mechanisms of Action. Pharmaceutics.

[5] Le J., Zhang L., Zhao W., Zhu S., Lan C., Kou J., Zhang Q., Zhang Y., Li Q., Chen Z. (2022). Infrequent Intranasal Oxytocin Followed by Positive Social Interaction Improves Symptoms in Autistic Children: A Pilot Randomized Clinical Trial. Psychother. Psychosom..

[6] Parker K.J., Oztan O., Libove R.A., Sumiyoshi R.D., Jackson L.P., Karhson D.S., Summers J.E., Hinman K.E., Motonaga K.S., Phillips J.M. (2017). Intranasal oxytocin treatment for social deficits and biomarkers of response in children with autism. Proc. Natl. Acad. Sci. USA.

[7] Yamasue H., Kojima M., Kuwabara H., Kuroda M., Matsumoto K., Kanai C., Inada N., Owada K., Ochi K., Ono N. (2022). Effect of a novel nasal oxytocin spray with enhanced bioavailability on autism: A randomized trial. Brain.

[8] Yatawara C.J., Einfeld S.L., Hickie I.B., Davenport T.A., Guastella A.J. (2016). The effect of oxytocin nasal spray on social interaction deficits observed in young children with autism: A randomized clinical crossover trial. Mol. Psychiatry.

[9] De Cagna F., Fusar-Poli L., Damiani S., Rocchetti M., Giovanna G., Mori A., Politi P., Brondino N. (2019). The Role of Intranasal Oxytocin in Anxiety and Depressive Disorders: A Systematic Review of Randomized Controlled Trials. Clin. Psychopharmacol. Neurosci..

[10] Bharadwaj V.N., Tzabazis A.Z., Klukinov M., Manering N.A., Yeomans D.C. (2021). Intranasal Administration for Pain: Oxytocin and Other Polypeptides. Pharmaceutics.

[11] Van Eyk A.D., Vand der Bijl P., Moll L.M. (2008). Physicochemical characteristics of molecules and their diffusion across human vaginal mucosa. Eur. J. Inflamm..

[12] Moreno-López Y., Martínez-Lorenzana G., Condés-Lara M., Rojas-Piloni G. (2013). Identification of oxytocin receptor in the dorsal horn and nociceptive dorsal root ganglion neurons. Neuropeptides.

[13] Warfvinge K., Krause D.N., Maddahi A., Grell A.S., Edvinsson J.C., Haanes K.A., Edvinsson L. (2020). Oxytocin as a regulatory neuropeptide in the trigeminovascular system: Localization, expression and function of oxytocin and oxytocin receptors. Cephalalgia.

[14] Yamamoto Y., Higashida H. (2020). RAGE regulates oxytocin transport into the brain. Commun. Biol..

[15] Martins D.A., Mazibuko N., Zelaya F., Vasilakopoulou S., Loveridge J., Oates A., Maltezos S., Mehta M., Wastling S., Howard M. (2020). Effects of route of administration on oxytocin-induced changes in regional cerebral blood flow in humans. Nat. Commun..

[16] Yao S., Chen Y., Zhuang Q., Zhang Y., Lan C., Zhu S., Becker B., Kendrick K.M. (2023). Sniffing oxytocin: Nose to brain or nose to blood?. Mol. Psychiatry.

[17] Maher S., Mrsny R.J., Brayden D.J. (2016). Intestinal permeation enhancers for oral peptide delivery. Adv. Drug Deliv. Rev..

[18] Hashim H., Abrams P. (2008). Desmopressin for the treatment of adult nocturia. Future Med. LTD.

[19] Maejima Y., Horita S., Otsuka A., Hidema S., Nishimori K., Shimomura K. (2020). Oral oxytocin delivery with proton pump inhibitor pretreatment decreases food intake. Peptides.

[20] Prego C., Garcia M., Torres D., Alonso M.J. (2005). Transmucosal macromolecular drug delivery. J. Control. Release.

[21] Frokjaer S., Otzen D.E. (2005). Protein drug stability: A formulation challenge. Nat. Rev. Drug Discov..

[22] Rathbone M.J., Drummond B.K., Tucker I.G. (1994). The oral cavity as a site for systemic drug delivery. Adv. Drug Deliv. Rev..

[23] Bastos F., Pinto A.C., Nunes A., Simões S. (2022). Oromucosal products—Market landscape and innovative technologies: A review. J. Control. Release.

[24] Mehta A.C. (1986). Buccal and oral drugs: Induction of labour. Acta. Chir. Hung..

[25] Westergaard J.G., Lange A.P., Pedersen G.T., Secher N.J. (1983). Oral oxytocics for induction of labor. A randomized study of prostaglandin E2 tablets and demoxytocin resoriblets. Acta. Obstet. Gynecol. Scand..

[26] Gleeson J.P., Fein K.C., Whitehead K.A. (2021). Oral delivery of peptide therapeutics in infants: Challenges and opportunities. Adv. Drug Deliv. Rev..

[27] Sharman A., Low J. (2008). Vasopressin and its role in critical care. Cont. Ed. Anaesth. Crit. Care Pain.

[28] van Kerrebroeck P., Nørgaard J.P. (2009). Desmopressin for the treatment of primary nocturnal enuresis. Pediatr. Health.

[29] Schiele J.T., Quinzler R., Klimm H.-D., Pruszydlo M.G., Haefeli W.E. (2013). Difficulties swallowing solid oral dosage forms in a general practice population: Prevalence, causes, and relationship to dosage forms. Eur. J. Clin. Pharmacol..

[30] (2020). FDA. https://www.fda.gov/safety/recalls-market-withdrawals-safety-alerts/ferring-us-issues-voluntary-nationwide-recall-ddavpr-nasal-spray-10-mcg01ml-desmopressin-acetate.

[31] Kottke D., Burckhardt B.B., Knaab T.C., Breitkreutz J., Fischer B. (2021). Development and evaluation of a composite dosage form containing desmopressin acetate for buccal administration. Int. J. Pharm. X.

[32] Hoffmann E.M., Breitenbach A., Breitkreutz J. (2011). Advances in orodispersible films for drug delivery. Exp. Op. Drug Deliv..

[33] Chen Y., Zou H., Hou X., Lan C., Wang J., Qing Y., Chen W., Yao S., Kendrick K.M. (2023). Oxytocin administration enhances pleasantness and neural responses to gentle stroking but not moderate pressure social touch by increasing peripheral concentrations. Elife.

[34] Kou J., Lan C., Zhang Y., Wang Q., Zhou F., Zhao Z., Montag C., Yao S., Becker B., Kendrick K.M. (2021). In the nose or on the tongue? Contrasting motivational effects of oral and intranasal oxytocin on arousal and reward during social processing. Transl. Psychiatry.

[35] Zhuang Q., Zheng X., Yao S., Zhao W., Becker B., Xu X., Kendrick K.M. (2022). Oral Administration of Oxytocin, Like Intranasal Administration, Decreases Top-Down Social Attention. Int. J. Neuropsychopharmacol..

[36] Pawar P.G., Darekar A.B., Saudagar R.B. (2018). Medicated chocolate and lollipops: A novel drug delivery system for pediatric patients. Pharma. Sci. Monitor..

[37] Shetty S., Kamath K., Shabaraya R., Miranda F.C. (2019). Design and development of medicated lollipop containing albendazole. Am. J. Pharmatech. Res..

[38] Tangso K.J., Ho Q.P., Boyd B.J. (2015). Confectionery-based dose forms. Curr. Drug Deliv..

[39] Gasmi Benahmed A., Gasmi A., Arshad M., Shanaida M., Lysiuk R., Peana M., Pshyk-Titko I., Adamiv S., Shanaida Y., Bjørklund G. (2020). Health benefits of xylitol. Appl. Microbiol. Biotechnol..

[40] Xu D., Li Q., Zhuang Q., Zhang Y., Yao S., Zhao W., Kendrick K.M. (2022). Oro-mucosal administration of oxytocin using medicated lollipops alters social attention, similar to intranasal and lingual routes: Implications for therapeutic use. Front. Neurosci..

[41] Daughters K., Manstead A.S.R., Hubble K., Rees A., Thapar A., van Goozen S.H.M. (2015). Salivary Oxytocin Concentrations in Males following Intranasal Administration of Oxytocin: A Double-Blind, CrossOver Study. PLoS ONE.

[42] van Ijzendoorn M.H., Bhandari R., van der Veen R., Grewen K.M., Bakermans-Kranenburg M.J. (2012). Elevated Salivary Levels of Oxytocin Persist More than 7 h after Intranasal Administration. Front. Neurosci..

[43] Weisman O., Zagoory-Sharon O., Feldman R. (2012). Intranasal oxytocin administration is reflected in human saliva. Psychoneuroendocrinology.

[44] Weisman O., Schneiderman I., Zagoory-Sharon O., Feldman R. (2013). Salivary vasopressin increases following intranasal oxytocin administration. Peptides.

[45] Martins D., Gabay A.S., Mehta M., Paloyelis Y. (2020). Salivary and plasmatic oxytocin are not reliable trait markers of the physiology of the oxytocin system in humans. Elife.

[46] Striepens N., Kendrick K.M., Hanking V., Landgraf R., Wüllner U., Maier W., Hurlemann R. (2013). Elevated cerebrospinal fluid and blood concentrations of oxytocin following its intranasal administration in humans. Sci. Rep..

[47] Kou J., Zhang Y., Zhou F., Sindermann C., Montag C., Becker B., Kendrick K.M. (2022). A randomized trial shows dose-frequency and genotype may determine the therapeutic efficacy of intranasal oxytocin. Psychol. Med..

[48] Spengler F.B., Schultz J., Scheele D., Essel M., Maier W., Heinrichs M., Hurlemann R. (2017). Kinetics and Dose Dependency of Intranasal Oxytocin Effects on Amygdala Reactivity. Biol. Psychiatry.

[49] Lieberz J., Scheele D., Spengler F.B., Matheisen T., Schneider L., Stoffel-Wagner B., Kinfe T.M., Hurlemann R. (2020). Kinetics of oxytocin effects on amygdala and striatal reactivity vary between women and men. Neuropsychopharmacology.

[50] Lan C., Chen Y., Zhang Y., Kou J., Huang L., Xu T., Yang X., Xu D., Yang W., Kendrick K.M. (2023). Oral Oxytocin Facilitates Responses to Emotional Faces in Reward and Emotional-Processing Networks in Females. Neuroendocrinology.

[51] Guo Y., Singh A.P. (2019). Emerging strategies for enhancing buccal and sublingual administration of nutraceuticals and pharmaceuticals. J. Drug Deliv. Sci. Tech..

[52] Mazzinelli E., Favuzzi I., Arcovito A., Castagnola R., Fratocchi G., Mordente A., Nocca G. (2023). Oral Mucosa Models to Evaluate Drug Permeability. Pharmaceutics.

[53] Nugrahadi P.P., Hinrichs W.L.J., Frijlink H.W., Schöneich C. (2023). Designing formulation strategies for enhanced stability of therapeutic peptides in aqueous solutions: A review. Pharmaceutics.

[54] Goyal D.K., Neil J.R., Simmons S.D., Mansab F., Benjamin S., Pitfield V., Boulet S., Miyan J. (2019). Zinc Deficiency in Autism: A Controlled Study. Insights Biomed..

[55] Alsufiani H.M., Alkhanbashi A.S., Laswad N.A.B., Bakhadher K.K., Alghamdi S.A., Tayeb H.O., Tarazi F.I. (2022). Zinc deficiency and supplementation in autism spectrum disorder and Phelan-McDermid syndrome. J. Neurosci. Res..

[56] Wu J., Wang D., Yan L., Jia M., Zhang J., Han S., Han J., Wang J., Chen X., Zhang R. (2022). Associations of essential element serum concentrations with autism spectrum disorder. Environ. Sci. Pollut. Res. Int..

[57] Liu D., Seuthe A.B., Ehrler O.T., Zhang X., Wyttenbach T., Hsu J.F., Bowers M.T. (2005). Oxytocin-receptor binding: Why divalent metals are essential. J. Am. Chem. Soc..

[58] Avanti C., Hinrichs W.L., Casini A., Eissens A.C., Van Dam A., Kedrov A., Driessen A.J.M., Frijlink H.W., Permentier H.P. (2013). The formation of oxytocin dimers is suppressed by the zinc-aspartate-oxytocin complex. J. Pharm. Sci..

[59] Khan K.S., Wojdyla D., Say L., Gülmezoglu A.M., Van Look P.F.A. (2006). WHO analysis of causes of maternal death: A systematic review. Lancet.

[60] Zhu C., Estrada M., White J., Lal M. (2018). Heat-stable sublingual oxytocin tablets as a potential needle-free approach for preventing postpartum hemorrhage in low-resource settings. Drug. Deliv. Transl. Res..

[61] Rossino G., Marchese E., Galli G., Verde F., Finizio M., Serra M., Linciano P., Collina S. (2023). Peptides as Therapeutic Agents: Challenges and Opportunities in the Green Transition Era. Molecules.

[62] Alghanem S., Dziurkowska E., Ordyniec-Kwaśnica I., Sznitowska M. (2023). Intraoral medical devices for sustained drug delivery. Clin. Oral. Investig..

[63] Bernstein G. (2008). Delivery of insulin to the buccal mucosa utilizing the rapidmist™ system. Expert. Opin. Drug. Deliv..

